# Deciphering the genetic basis of electrolyte homeostasis: genome-wide association studies of plasma sodium, chloride, and potassium in two European cohorts

**DOI:** 10.1093/ndt/gfag069

**Published:** 2026-03-24

**Authors:** Mirko Treccani, Anna Cornelia van der Burgh, Gianmarco Lombardi, Mohsen Ghanbari, Ewout Jasper Hoorn, Giovanni Malerba, Giovanni Gambaro, Pietro Manuel Ferraro

**Affiliations:** Department of Surgical Sciences, Dentistry, Paediatrics and Gynaecology, University of Verona, Verona, Italy; Department of Epidemiology, Erasmus MC, University Medical Center Rotterdam, Rotterdam, The Netherlands; Section of Nephrology, Department of Medicine, University of Verona, Verona, Italy; Department of Epidemiology, Erasmus MC, University Medical Center Rotterdam, Rotterdam, The Netherlands; Department of Internal Medicine, Division of Nephrology and Transplantation, Erasmus MC, University Medical Center Rotterdam, Rotterdam, The Netherlands; Department of Surgical Sciences, Dentistry, Paediatrics and Gynaecology, University of Verona, Verona, Italy; Section of Nephrology, Department of Medicine, sUniversity of Verona, Verona, Italy; Section of Nephrology, Department of Medicine, University of Verona, Verona, Italy

**Keywords:** genome-wide association studies, kidney physiology, plasma electrolytes, population-based study

## Abstract

**Background and hypothesis:**

Chloride, potassium, and sodium are fundamental electrolytes involved in key kidney physiological processes. Previous studies focusing on the genetics of electrolyte homeostasis have mainly investigated their urinary concentrations. To date, only a few studies have investigated the genetic factors influencing the differences in plasma levels of electrolytes in the general population.

**Methods:**

In this study, we performed genome-wide association studies (GWAS) followed by meta-analyses and post-GWAS computational investigations on the circulating levels of these electrolytes in two European cohorts, the INCIPE and the Rotterdam Study (overall *n* = 9 256).

**Results:**

We identified 11 significant variants in eight independent loci surpassing the genome-wide significance threshold (*P* < 5 × 10^−8^) for chloride, along with 88, 21, and 24 genomic loci suggestively associated (*P* < 1 × 10^−5^) with sodium, chloride, and potassium levels, respectively. Post-GWAS computational analyses on associated genes and variants suggested differential regulation of gene expression in heart, muscle, and kidney tissues, as well as enrichment for gene sets related to positional, transcription factor target and cell type signatures.

**Conclusions:**

The findings of this study highlight a significant genetic contribution to the homeostasis of plasma chloride, potassium, and sodium concentrations, and confirm that these electrolytes are involved not only in kidney physiology but also in broader biological processes and complex systemic interactions in the human body.

## Graphical Abstract

**Graphical Abstract:**
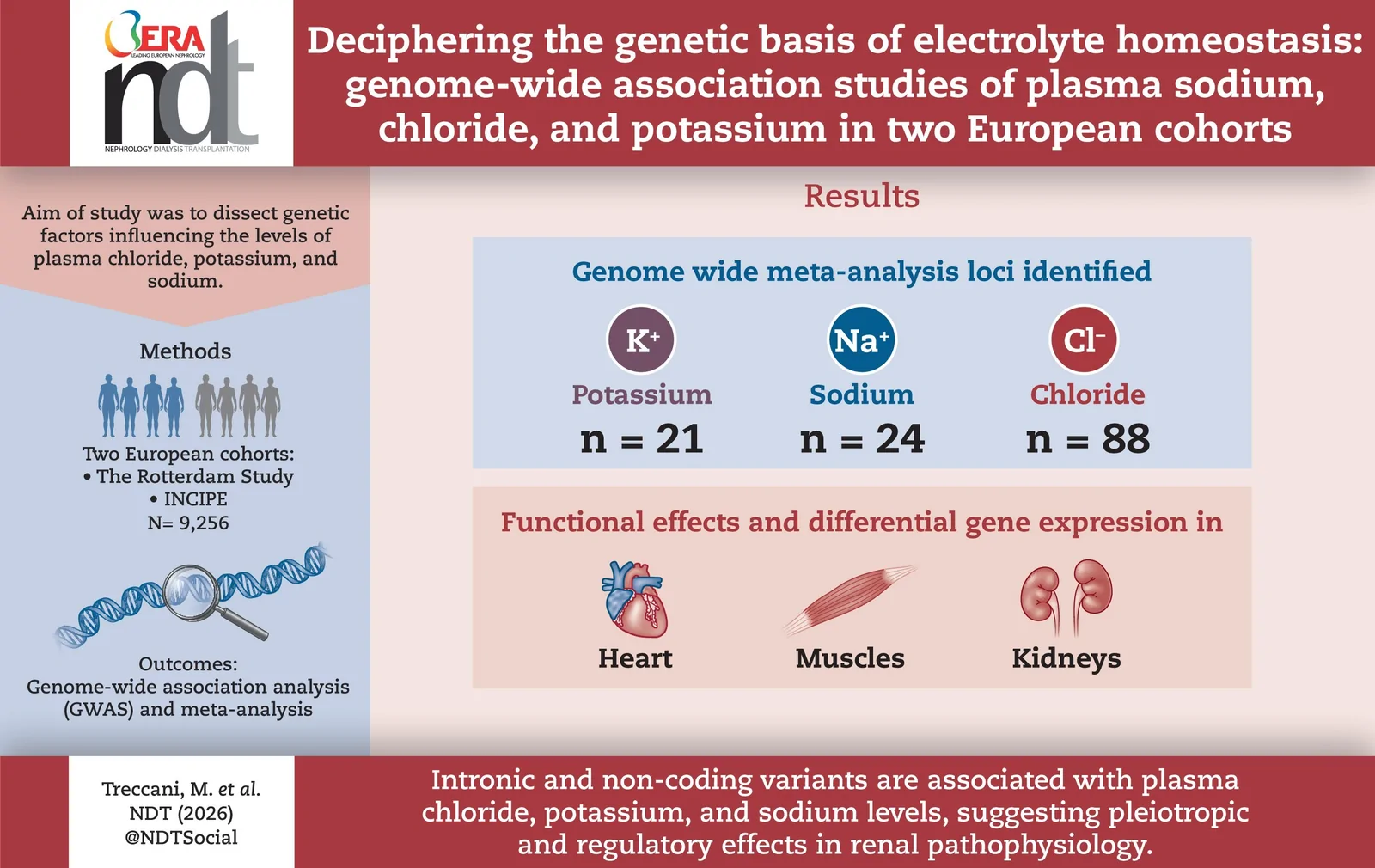

KEY LEARNING POINTS **What was known:**

- Genome-wide meta-analysis in two European cohorts identifying 24, 88, and 21 loci associated with plasma sodium, chloride, and potassium levels.

**This study adds:**

- Computational functional analysis showing significant enrichment in positional gene sets, suggesting local regulatory effects that need to be further explored.

**Potential impact:**

- Tissue specificity analysis showing a significantly increased expression in heart and muscle tissues and a nominal significance in kidney tissues.

## INTRODUCTION

Electrolytes such as chloride (Cl), potassium (K), and sodium (Na) are essential players in several biological processes [1]. These electrolytes are involved in similar and interconnected functions: e.g. plasma sodium reflects total body water and maintains fluid balance and plasma volume, being an expression of the total body water; plasma potassium maintains membrane potential; and plasma chloride is involved in acid-base status; overall, all contribute to plasma osmolality. Their concentrations are regulated by the kidney, the pituitary, and the adrenal glands. Electrolyte imbalances are associated with a variety of acute and chronic diseases. They can serve not only as hallmarks of conditions such as chronic kidney disease (CKD), acid-base disorders, and endocrine disorders, but also as factors linked to mortality risk, often demonstrating a U-shaped relationship with plasma electrolyte levels [2–5]. In the era of precision medicine, the inter-individual variability in plasma electrolyte levels remains only partially understood and needs to be further investigated for the development of tailored therapeutic strategies. Different studies have investigated whether genetic factors influence electrolyte levels and their impact on kidney health and diseases [6–8]. However, most of these studies have focused on urinary electrolyte excretions, with little attention given to plasma levels. The genetic contribution to plasma chloride, potassium, and sodium has been only partially investigated, generally through a candidate gene approach [6] or focusing on specific pathological conditions [7]. Recently, a large-scale genome-wide association study (GWAS) was performed on plasma sodium to investigate the genetics of plasma concentration and exposure to diuretics, reporting many associated loci [8]. In the current study, we aimed to dissect genetic factors influencing the levels of plasma chloride, potassium, and sodium in the general population. We performed GWAS followed by meta-analyses in two European cohorts: the “Initiative on Nephropathy, of relevance to public health, which is Chronic, possibly in its Initial stages, and carries a Potential risk of major clinical End-points” (INCIPE) [9] and the Rotterdam Study (RS) [10, 11]. For the identified variants and loci associated with plasma chloride, potassium, and sodium levels, further computational analyses were conducted to support their potential function in relation to kidney health and disease.

## MATERIALS AND METHODS

### The study population

The INCIPE enrolled individuals aged 40+ years to investigate CKD prevalence in the Northeast area of Italy. The sampling of participants from general medical practices ensured generalizability of the study population to the general adult population of the area, by virtue of the organization of the health system in Italy. Enrolled individuals underwent a standardized collection of medical history and physical examination, and collection of blood and urine samples for further analysis.

The RS is a prospective population-based cohort study in the Netherlands, among inhabitants of a well-defined suburb of Rotterdam (Ommoord), with the aim of describing the prevalence and incidence, unraveling the etiology, and identifying targets for prediction, prevention, or intervention of multifactorial diseases in mid-life and the elderly. A comprehensive description of the study design has been published previously [10, 11]. Briefly, during the first recruitment wave in 1990 (RS-I), all inhabitants from Ommoord aged ≥55 years were invited for participation, of whom 7 983 consented (response rate: 78%). During the second recruitment wave in 2000 (RS-II) and using the same criteria, the cohort was expanded with 3 011 individuals (response rate: 67%). By the third recruitment wave in 2006 (RS-III), individuals aged ≥45 years were invited as well, upon which 3 932 consented (response rate: 65%). The RS is still ongoing and, with the most recent extension of the cohort (RS-IV), comprises more than 17 931 participants aged 45 years or over, who have follow-up examinations every 3–5 years.

In both cohorts, plasma chloride, potassium, and sodium levels were measured using ion-selective electrodes and were quantified using standard measurement units (mmol/l).

Genetic information was retrieved by genome-wide genotyping using the Illumina Human Omni Express (INCIPE1) and the Illumina Human Core Exome (INCIPE2) arrays in the INCIPE cohorts, while in the RS cohort, the genotyping was conducted using Illumina 550 K (RS-I-3 and RS-II-1) and 610 K (RS-III-1) Affymetrix arrays.

### Genomic associations and meta-analyses

After genotyping, the two cohorts underwent quality controls using PLINK 1.90 [12], considering the following exclusion criteria: genotype missingness >0.02, sample missingness >0.15, discordant clinical and genetic sexual information, first- and second-degree relatives, and non-European genetic ancestries. Related individuals were identified using kinship coefficients. Genetic ancestry was determined using principal component (PC) analysis, after pruning regions in linkage disequilibrium with a window size of 50 kb, the variant count >10, and pairwise variant correlation (r^2^) >0.2. Genotype imputation [13] was performed using Minimac4 [14] on the European sample from the Human Reference Consortium (HRC) panel [15]; phasing was performed using Eagle 2.4 [16] and the imputation quality threshold was set to *R*^2^ >0.6.

GWAS was performed for each electrolyte using Rvtest [17], considering the dosage information, excluding rare variants [minor allele frequency (MAF) >0.01], and correcting for sex, age, and the first five PCs. Significance thresholds were set as suggestive at *P* = 1 × 10^−5^ and genome-wide at *P* = 5 × 10^−8^. GWAS summaries across the five subcohorts (INCIPE1, INCIPE2, RS-I-3, RS-II-1, and RS-III-1) were combined and meta-analyzed using the sample size scheme available in METAL [18]: concordant significant signals and showing missing information in at most two subcohorts were retained. Genetic findings were searched on the GWAS Catalog version e0_r2022-11-29 [19] to query for already reported associations.

### Computational functional analysis and annotation

Computational functional analysis was performed to understand the possible biological functions related to the associated signals. The SNP2GENE and GENE2FUNC modules from FUMAGWAS v1.5.2 [20] were employed, using ANNOVAR version 2017-07-17 [21] for variant annotation, MAGMA version 1.08 [22] for gene sets analysis on the Genotype-Tissue Expression (GTEx) project version 8 [23] and the Molecular Signature database (MSigDB) [24] data. Resulting *P*-values were adjusted according to the Bonferroni correction method. Nominal significance was set to *P* = .05.

## RESULTS

### Characteristics of participants: the INCIPE cohort

The INCIPE cohort comprised 3 064 individuals and consisted of two subcohorts, namely INCIPE1 (940 individuals and 650 655 genetic markers) and INCIPE2 (2 117 individuals and 534 065 genetic markers). After quality controls, a total of 912 samples and 645 475 markers and 1 933 samples and 477 561 markers were retained in INCIPE1 and INCIPE2, respectively. Furthermore, for each electrolyte, only individuals having a non-missing phenotype were retained: chloride and sodium were available in 857 (INCIPE1) and 1 343 (INCIPE2) subjects, and potassium in 853 (INCIPE1) and 1 341 (INCIPE2). Both the subsets presented almost similar age and sex distribution; plasma electrolyte concentrations were also similar, with slightly higher values in the INCIPE2 subcohort. After genotyping and quality controls, the two subcohorts underwent genotype imputation and subsequent filtering, empowering the available genetic information to 8 866 985 and 7 082 198 variants in INCIPE1 and INCIPE2, respectively. An overview is reported in Table 1.

**Table 1:** Overview of the INCIPE and RS cohorts.

	INCIPE1	INCIPE2	RS-I-3	RS-II-1	RS-III-1
Samples (*n*)	857	1 343	2 056	2 042	2 958
Typed and imputed genetic variants (*n*)	8 866 985	7 082 198	7 746 462	7 745 860	7 747 213
Sex (% men)	403 (47%)	631 (47%)	787 (38%)	935 (46%)	1296 (44%)
Age (mean ± sd) [years]	61.0 ± 11.5	59.7 ± 11.3	73.10 ± 7.65	64.84 ± 7.99	57.30 ± 6.89
Chloride (mean ± sd) [mmol/l]	102.3 ± 3.3	104.0 ± 2.9	100.82 ± 2.94	101.98 ± 2.43	102.34 ± 2.29
Potassium (mean ± sd) [mmol/l]	3.99 ± 0.38	4.02 ± 0.38	4.46 ± 0.37	4.29 ± 0.28	4.40 ± 0.30
Sodium (mean ± sd) [mmol/l]	137.2 ± 3.6	139.0 ± 2.9	141.16 ± 2.48	142.18 ± 2.13	141.41 ± 1.82

The table shows information on samples and phenotypes in the INCIPE1, INCIPE2, RS-I-3, RS-II-1, and RS-III-1 subcohorts.

### Characteristics of participants: the RS cohort

The RS cohort investigated in this study comprised the baseline data from the first three subcohorts, namely RS-I-3 (or ERGO3, 4 797 individuals), RS-II-1 (or ERGOPLUS, 3 011 individuals), and RS-III-1 (ERGOJONG, 3 932 individuals). After quality control and phenotype inclusion, a total of 2042 and 2958 individuals were retained for RS-II-1 and RS-III-1; instead, RS-I-3 presented 2056, 2035, and 2034 individuals for chloride, potassium and sodium, respectively. Next, genotype imputation leveraged the available genetic information, reaching 7 746 462, 7 745 860, and 7 747 213 variants in RS-I-3, RS-II-1, and RS-III-1, respectively. An overview is provided in Table 2.

**Table 2:** Top significant associations in the GWAS meta-analysis in the INCIPE and RS cohorts for chloride, potassium, and sodium plasma concentrations.

Chrom	Pos (bp)	Allele 1 (effect allele)	Allele 2	Nearest gene	Z-score	*P*-value
Chloride						
19	1 490 143	T	C	PCSK4	7.086	1.38 × 10^−12^
19	1 389 195	T	C	NDUFS7	−6.579	4.74 × 10^−11^
16	33 296 376	A	G	Intergenic	6.379	1.79 × 10^−10^
8	143 157 397	T	C	RP11-953B20.1	6.329	2.47 × 10^−10^
6	137 898 323	T	C	Intergenic	6.301	2.96 × 10^−10^
6	161 314 984	T	C	RP11-235G24.1	−5.969	2.39 × 10^−9^
18	56 629 776	A	G	ZNF532	−5.936	2.92 × 10^−9^
3	177 761 313	A	T	Intergenic	−5.929	3.06 × 10^−9^
6	138 061 647	C	G	Intergenic	−5.894	3.77 × 10^−9^
22	26 422 201	A	G	MYO18B	−5.802	6.57 × 10^−9^
Potassium						
3	163 730 005	A	T	RP11-208P4.1	−5.219	1.80 × 10^−7^
5	137 231 799	C	G	KLHL3	−5.214	1.85 × 10^−7^
5	137 229 414	C	G	PKD2L2	−5.211	1.88 × 10^−7^
5	137 219 692	T	C	MYOT	5.182	2.19 × 10^−7^
5	137 225 896	C	G	PKD2L2	5.172	2.32 × 10^−7^
5	137 241 856	T	C	PKD2L2	5.170	2.34 × 10^−7^
3	163 916 785	T	C	MIR1263	−5.145	2.67 × 10^−7^
5	137 228 063	T	C	PKD2L2	−5.137	2.79 × 10^−7^
5	137 221 011	A	T	MYOT	5.125	2.97 × 10^−7^
5	137 233 790	A	G	PKD2L2	5.117	3.11 × 10^−7^
Sodium						
15	48 265 323	T	C	RP11-208K4.1	5.481	4.22 × 10^−8^
15	48 239 588	A	G	RP11-208K4.1	5.393	6.94 × 10^−8^
11	101 225 319	A	G	TRPC6	−4.901	9.53 × 10^−7^
13	24 269 820	A	G	Intergenic	−4.899	9.61 × 10^−7^
20	55 276 252	A	G	PTMAP6	4.832	1.35 × 10^−7^
15	48 376 241	T	C	RP11-208K4.1	−4.821	1.43 × 10^−7^
20	38 875 017	A	G	Intergenic	−4.767	1.87 × 10^−7^
2	88 024 591	T	C	AC093616.4	4.759	1.94 × 10^−7^
3	135 696 782	A	G	PPP2R3A	4.750	20.3 × 10^−7^
3	84 564 209	C	G	AC107025.1	4.730	2.24 × 10^−7^

Chromosome and position refer to the human genome release hg19/GRCh37. Chrom: chromosome number; Pos: position (in base pair from the start for the chromosome).

### Genome-wide meta-analysis

After meta-analyses, genome-wide significant associations (*P* < 5 × 10^−8^) were identified for chloride, with a total of eight independent signals. Suggestive associations (*P* < 1 × 10^−5^) were identified for all three electrolytes, including 459, 131, and 50 variants for chloride, potassium, and sodium, respectively. An overview is reported in Fig. 1 and Table 2.

**Figure 1:**
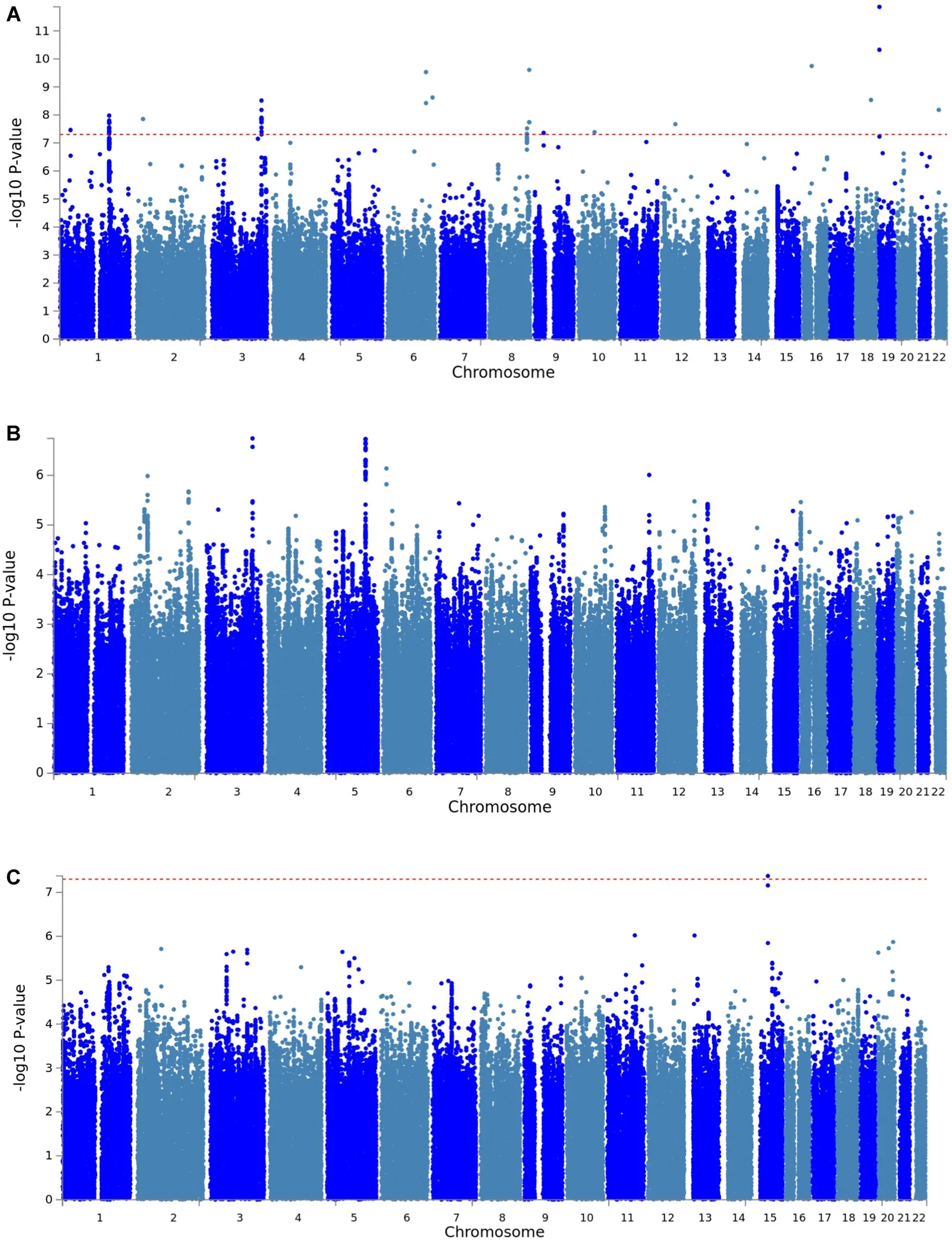
Manhattan plot from GWAS meta-analysis across the INCIPE and RS cohorts for the investigated electrolytes. Genetic associations with the plasma levels of (A) chloride, (B) potassium, and (C) sodium in the INCIPE and RS cohorts. Genome-wide significant threshold (horizontal dashed lines) was set to *P* = 5 × 10^−8^).

In terms of identified genomic loci, we found 88 loci for plasma chloride led by 89 independent SNPs (top significant), and 1 560 correlated (all significant) variants (LD > 0.6); plasma potassium displayed 21 loci and 21 independent leading and 1 560 correlated variants; plasma sodium showed 24 genomic loci and 24 independent leading and 216 correlated variants. More information is available in Supplementary Figures S1, S2, and S3, respectively.

Genomic annotation of genome-wide and suggestively associated variants showed that they were predominantly located in intergenic (Cl, *P* = 5.47 × 10^−1^; K, *P* = 1.81 × 10^−16^; Na, *P* = 8.73 × 10^−11^), intronic (Cl, *P* = 2.29 × 10^−5^; K, *P* = 1.85 × 10^−9^; Na, *P* = 2.19 × 10^−3^), and non-coding RNA-related regions (Cl, *P* = 3.21 × 10^−23^; K, *P* = 8.67 × 10^−5^; Na, *P* = 1.09 × 10^−5^). These categories were significantly over-represented among associated loci relative to the GWAS background and were consistently observed across all investigated electrolytes, as shown in Fig. 2.

**Figure 2:**
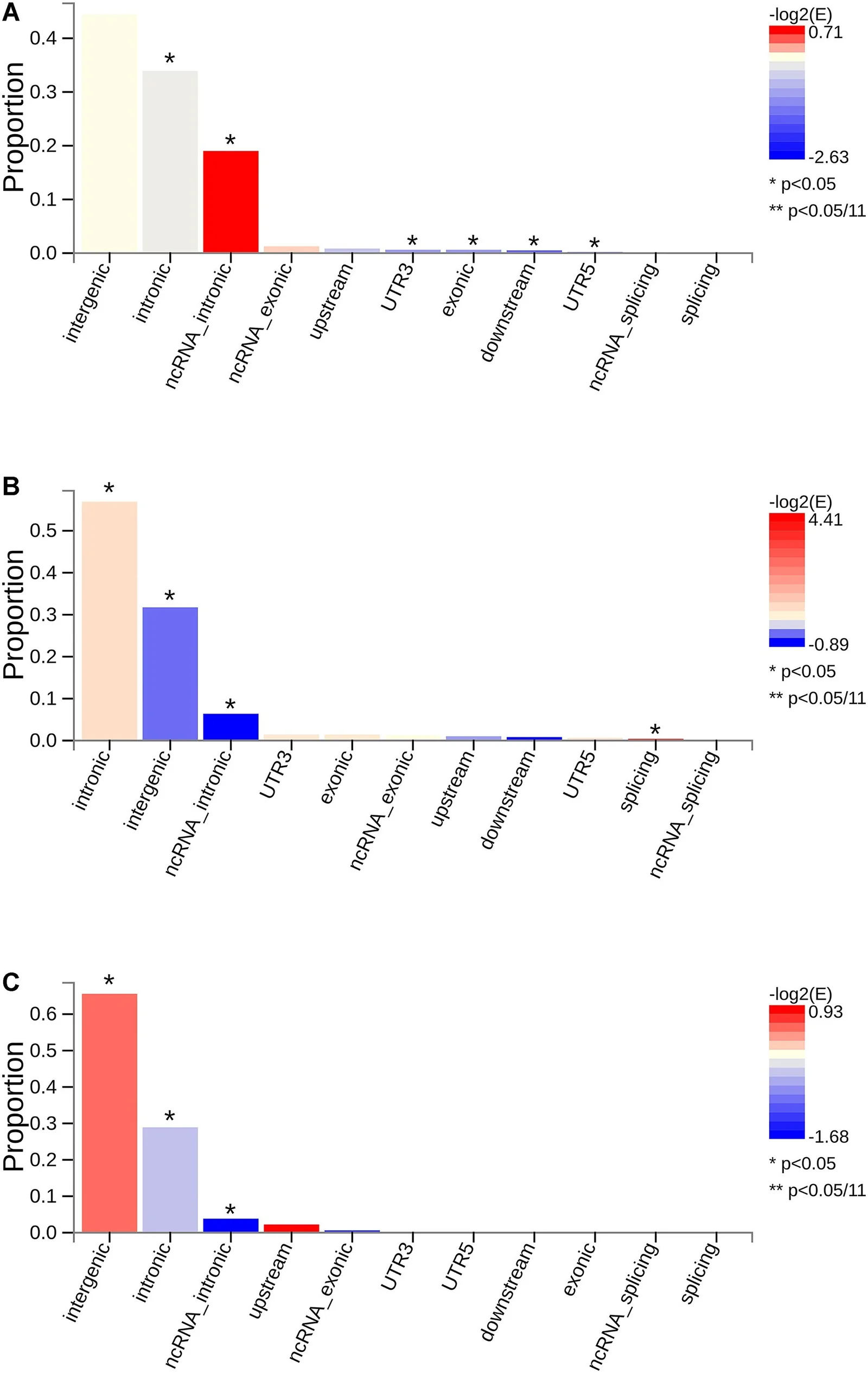
Annotation of associated variants across GWAS meta-analyses. The bar plots report the annotation of the associated variants with plasma levels of (A) chloride, (B) potassium, and (C) sodium in the INCIPE and RS cohorts. Significance threshold was set to *P* < .05. ncRNA: noncoding RNA.

For all significantly and suggestively associated variants, we searched in the GWAS Catalog for already reported associations with any other trait or disease. Relevant reported associations were observed for plasma chloride and potassium; the most relevant terms for plasma chloride referred to blood protein levels (*P* = 3 × 10^−70^), and then several carnitine metabolites (the most significant being isobutyrylcarnitine, *P* = 2 × 10^−34^), diastolic and systolic blood pressure (*P* = 1 × 10^−13^), and cystatin C levels (*P* = 2 × 10^−12^); for plasma potassium, the most relevant terms were related to lipids, such as triglycerides (*P* = 5 × 10^−305^), cholesterol (*P* = 6 × 10^−300^), and apolipoprotein B (APOB, *P* = 9 × 10^−138^) and A5 (APOA5, *P* = 4 × 10^−41^), and vitamins, such as vitamin D (*P* = 3 × 10^−14^) and 25-hydroxyvitamin D (*P* = 2 × 10^−50^). No relevant reported associations were observed for plasma sodium.

### Computational functional analysis

Computational functional analyses were performed on genome-wide and suggestive significant (*P* < 1 × 10^−5^) results from each of the three meta-analyses.

Gene-sets and tissue expression analyses showed no significant enrichment or association for any of the three electrolytes. However, a trend for association was observed in the pituitary tissue for plasma sodium (*P* = .05, Figure S4). Additionally, enriched data showed nominal significance (not adjusted *P*-value: *P* < .05) in chloride for ABC-mediated transport (*P* = 4.56 × 10^−5^) and L-type voltage-gated calcium channel complex (*P* = 3.29 × 10^−4^, Table S1).

Sets of genetic variants within a gene were simultaneously considered to perform a gene-based association analysis, increasing the overall discriminant power. Over 7 million typed and imputed variants were mapped on 18 771 protein-coding genes to perform gene-based association tests (significance threshold: *P* = 2.66 × 10^−6^). Significant associations were observed for potassium, on the MYOT (*P* = 1.91 × 10^−7^) and ZNF259 (*P* = 1.64 × 10^−6^, Fig. 3). No gene-based significant associations were observed for plasma chloride or plasma sodium.

**Figure 3:**
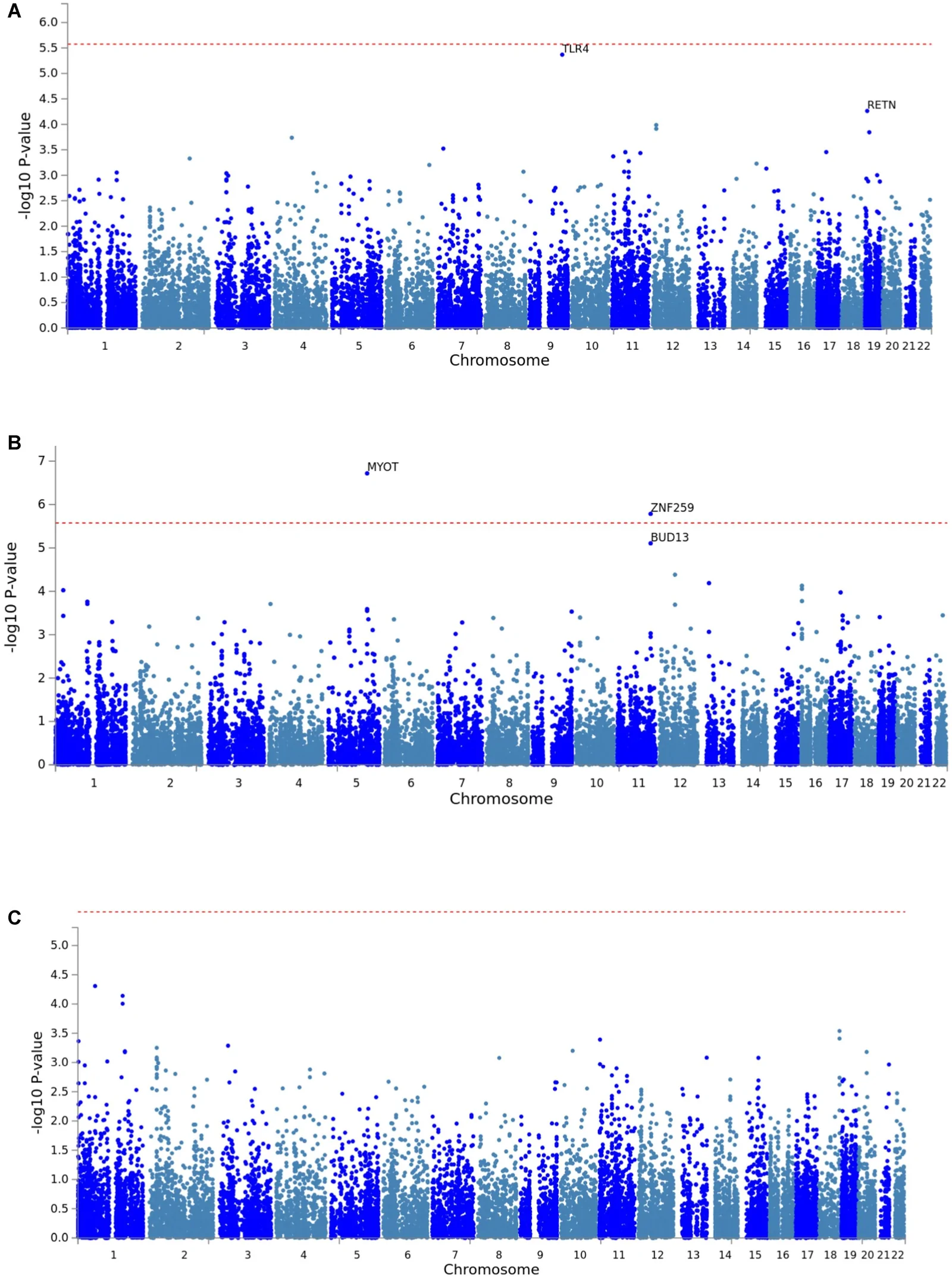
Manhattan plots from gene-wise association analysis across the INCIPE and Rotterdam Study cohorts for the investigated electrolytes chloride (A), potassium (B), and sodium (C). Significant and suggestive associated genes are reported with their symbols. Significance threshold (horizontal dashed line) was set to *P* = 2.66 × 10^−6^.

Differentially expressed genes across the general and specific tissue types from GTEx were subsequently investigated. In plasma chloride, nominal associations were observed in down-regulated genes from “kidney” (*P* = 6.79 × 10^−3^) and “kidney cortex” (*P* = 2.95 × 10^−3^) when compared to the other tissues. For plasma potassium, significant associations were observed for down regulated genes in “muscle” (*P* = 2.72 × 10^−4^; adjusted *P* = 8.16 × 10^−3^) and “skeletal muscle” (*P* = 2.72 × 10^−4^; adjusted *P* = 1.47 × 10^−2^); additionally, both-sided expressed genes were also significant in “heart” (*P* = 1.56 × 10^−3^; adjusted *P* = 4.69 × 10^−2^) and “heart atrial appendage” (*P* = 1.32 × 10^−4^; adjusted *P* = 7.16 × 10^−3^); “kidney” and “kidney cortex” reached nominal significance in down regulated genes (*P* = 1.46 × 10^−2^ and *P* = 1.2 × 10^−2^, respectively). For plasma sodium, nominal significance was observed for both-sided expressed genes in “kidney” (*P* = 4.30 × 10^−2^) and “kidney cortex” (*P* = 4.89 × 10^−2^). An overview is reported in Fig. 4 (general tissue types) and in Figures S5–S7 (specific tissue types).

**Figure 4:**
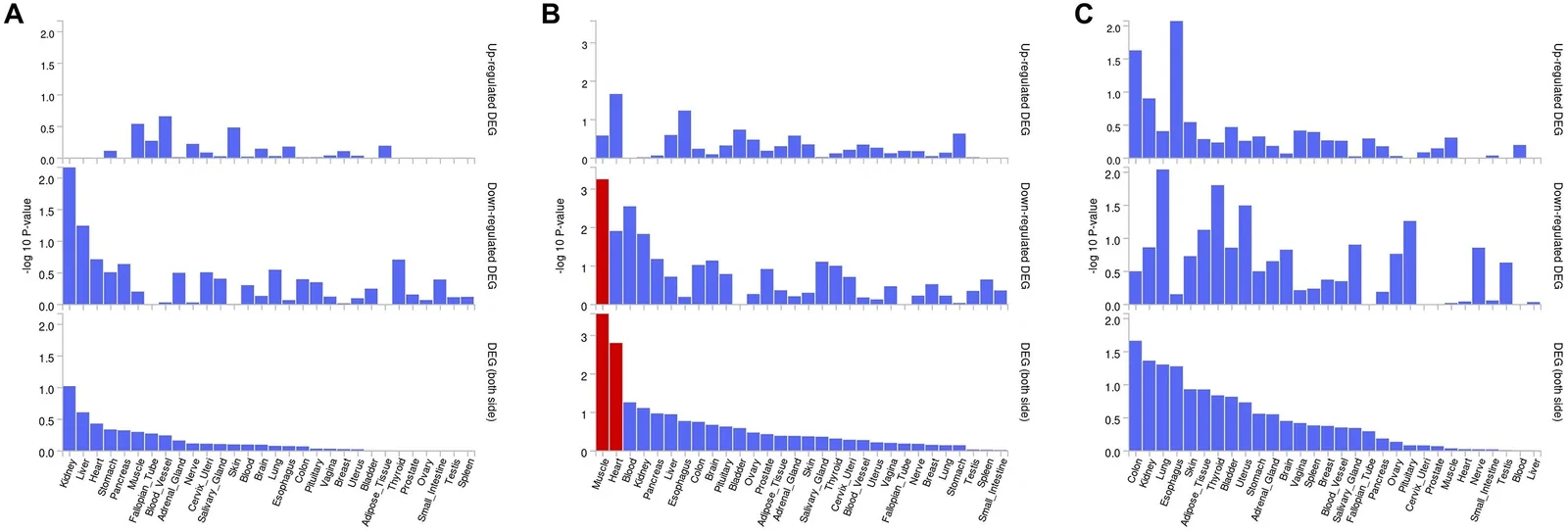
MAGMA expression analysis on the general tissue types from the Genotype-Tissue Expression portal. Bar plots from the expression level across the 30 general tissue types in the GTEx portal observed for (A) chloride, (B) potassium, and (C) sodium plasma levels. Significantly enriched tissue (Bonferroni-adjusted *P*-value <.05) are reported with red bars.

Next, gene set enrichment analyses were performed to further investigate the possible biological mechanisms related to serum electrolyte levels. For all the electrolytes, significant associations in positional gene sets were identified: 10 for plasma chloride, 6 for plasma potassium, and 3 for plasma sodium. Chromosomes 1, 2, 5, 9, and 11 were targeted for more than one electrolyte. No chromosomal positions were shared among electrolytes; however, proximal locations between plasma chloride and plasma potassium were found to be significant across 11q25 (*P* = 1.25 × 10^−5^) and 11q23 (*P* = 4.17 × 10^−6^), 2q31 (*P* = 2.68 × 10^−3^) and 2q33 (*P* = 3.76 × 10^−7^), and 5q33 (*P* = 8.52 × 10^−3^) and 5q31 (*P* = 3.29 × 10^−10^), respectively. Supporting evidence is reported in Table S2. The regional effects from the positional gene sets were further observed in the association of transcription factor (TF) targets: ZNF175 (*P* = 2.41 × 10^−−2^) for plasma chloride; CEBP01 (*P* = 1.54 × 10^−2^), CEBPZ (*P* = 4.31 × 10^−2^), and ZNF134 (*P* = 4.31 × 10^−2^) for plasma potassium. Additionally, plasma potassium also showed associations for pathways related to statin inhibition of cholesterol production (*P* = 1.65 × 10^−2^) and cholesterol metabolism (*P* = 1.65 × 10^−2^). Additionally, specific kidney cell type signatures were significantly enriched for plasma sodium, including distal convoluted tubule (*P* = 4.28 × 10^−3^) and thick ascending limb (*P* = 7.77 × 10^−3^). Supporting evidence is reported in Table S3.

## DISCUSSION

In this study, we conducted a genomic investigation on the levels of three plasma electrolytes (sodium, chloride, and potassium) in two cohorts of European ancestry. Rather than categorizing plasma electrolytes according to dichotomous thresholds (e.g. hyponatremia, hyperkalemia), our study investigated their continuous plasma concentrations, aiming to explore their biological complexity and variability through a genetic lens.

Genetic associations with plasma chloride levels resulted in the overall most significant results among the three electrolytes, identifying eight distinct genome-wide significant peaks. Among these loci, genes and genetic regions are reported to be involved in inflammatory and innate immunity pathways (such as TLR4 and IL1RAP) or in intracellular trafficking (such as DYNC1I2 and KIF2A), suggesting their possible involvement in chloride homeostasis [25–27]. Plasma potassium levels were mostly associated with genes related to signal transduction (such as PLCB1 and PRKAG2), and protein degradation (such as FANCL and PSMD12). Among these, PRKAG2 (Protein Kinase AMP-Activated Non-Catalytic Subunit Gamma 2) and PLCB1 (Phospholipase C Beta 1) might influence plasma potassium levels through their roles in cellular signaling pathways [28] and energy metabolism [29–31], respectively. Mutations in the PRKAG2 gene are associated with pathophysiological heart conditions [32]. The association with MYOT (Myotilin) may indicate a role of skeletal muscle structure in long-term potassium buffering and potassium plasma levels. Skeletal muscle accounts for approximately 70% of total body potassium and constitutes the largest intracellular reservoir involved in potassium buffering and distribution. Myotilin confers structural integrity to the sarcomeres at the Z-discs in striated muscle; its mutations cause myopathies and cardiomyopathies [33]. The significant association of plasma potassium levels with ZNF259 and the suggestive significance of BUD13, together with the enrichment of pathways related to statin inhibition of cholesterol production and metabolism, point to a role of these genes and potassium regulation in dyslipidemia [34].

Despite potential biological relevance, plasma sodium concentration yielded few genome-wide significant results. TRPC6 (Transient Receptor Potential Cation Channel Subfamily C Member 6) encodes for a cation channel involved in glomerular and tubular signaling [35, 36], CYP24A1 (Cytochrome P450 Family 24 Subfamily A Member 1) is involved in vitamin D metabolism, and RAPGEF1 (Rap Guanine Nucleotide Exchange Factor 1) influences epithelial sodium channel (ENaC) activities [37].

All the electrolytes were enriched in terms of significant positional gene sets, sharing regions in close proximity likely to influence electrolytes’ homeostasis. These results highlight genomic regions where associated variants tend to cluster, suggesting possible local effects on the balance of these electrolytes, including regulatory effects, dosage compensation, and epigenetic mechanisms worth investigating with additional functional studies [38, 39]. These effects were further explored using tissue expression data from the GTEx catalog. Studies have shown that some disease-causing variants appear to be enriched in disorder-relevant cell types [39]. Expression in the striated muscle and heart resulted in significant levels of potassium, most likely due to its physiological effects on contraction, likely supporting the putative role of PRKAG2 and MYOT in the regulation of plasma potassium levels [40, 41]. Of note, consistently with the recent GWAS from Andersson and colleagues [8], genes associated with plasma sodium were mostly expressed in the pituitary tissue. Regarding the kidneys, suggestive associations were reached in all three electrolytes for down-regulation in the kidney and kidney cortex tissues, supporting the biological relevance of the findings. This trend was further confirmed for plasma sodium, where cell signatures belonging to specific kidney compartments, such as the distal convoluted tubule and the thick ascending limb, were significantly enriched [42].

This study has several limitations. Despite investigating five different subcohorts from two main European cohorts, the sample size may be a limiting factor in obtaining robust results due to the expected small effect size of multiple genetic variants, as well as the possible contribution of rare variants to the plasma profiles of interest. Although the heterogeneity in the genetic background among the cohorts has been controlled for by correcting for sex, age, and PCs. Such corrections may have reduced the statistical power of our analysis, as well as not fully addressed the possible genetic heterogeneity in Northern and Southern Europeans. In addition, the present findings cannot be directly generalized to populations of non-European ancestry.

Overall, our study identified several associations beyond the kidneys, highlighting common genetic variants predominantly in intronic and non-coding regions, as typically observed for complex traits [43–46]. The present data also reveal novel biological pathways linking plasma electrolyte regulation to several organs and tissues—namely muscle and heart—as well as inflammation and cellular transduction and energetic mechanisms. Additionally, shared loci and positional hotspots were observed, which may suggest shared biological functions, possible pleiotropic effects, and *cis-*regulatory clustering in kidney electrolyte genes. These findings provide novel insights into the genetic architecture underlying the homeostasis and regulation of these electrolytes. Expanding GWAS cohorts and meta-analyses will strengthen signal detection and validation; moreover, network and 3D genomics [47] approaches may help disentangle the genetic complexity of plasma electrolytes and explain the role of non-coding and regulatory variants in kidney pathophysiology.

## Supplementary Material

gfag069_Supplemental_Files

## Data Availability

Genetic data from the INCIPE and the Rotterdam Study cohorts are available upon request due to privacy regulations and informed consent of the participants.
