# Supplementary material for: Deciphering the genetic basis of electrolyte homeostasis: genome-wide association studies of plasma sodium, chloride, and potassium in two European cohorts
Source: Nephrol Dial Transplant. 2026 Mar 24;41(11):2008–18. doi: 10.1093/ndt/gfag069 (PMC13719705; doi:10.1093/ndt/gfag069)

Supplemental Materials for "Deciphering the genetic basis of electrolyte homeostasis: Genome-Wide Association Studies of plasma sodium, chloride, and potassium in two European cohorts" by Mirko Treccani et al.

Table of contents

Supplemental Figures

Supplemental Figure 1..page 2

Supplemental Figure 2..page 3

Supplemental Figure 3..page 4

Supplemental Figure 4..page 5

Supplemental Figure 5..page 6

Supplemental Figure 6..page 7

Supplemental Figure 7..page 8

Supplemental Tables

Supplemental Table 1, Supplemental Table 2, and Supplemental Table 3 are available as spreadsheet documents

Supplemental Figure 1. Significant genomic loci associated with plasma chloride levels. The bar plot shows the significant genomic loci with their size in kilobases (kb), the number of SNPs and of mapped genes within each locus, and the number of genes physically located in each locus.

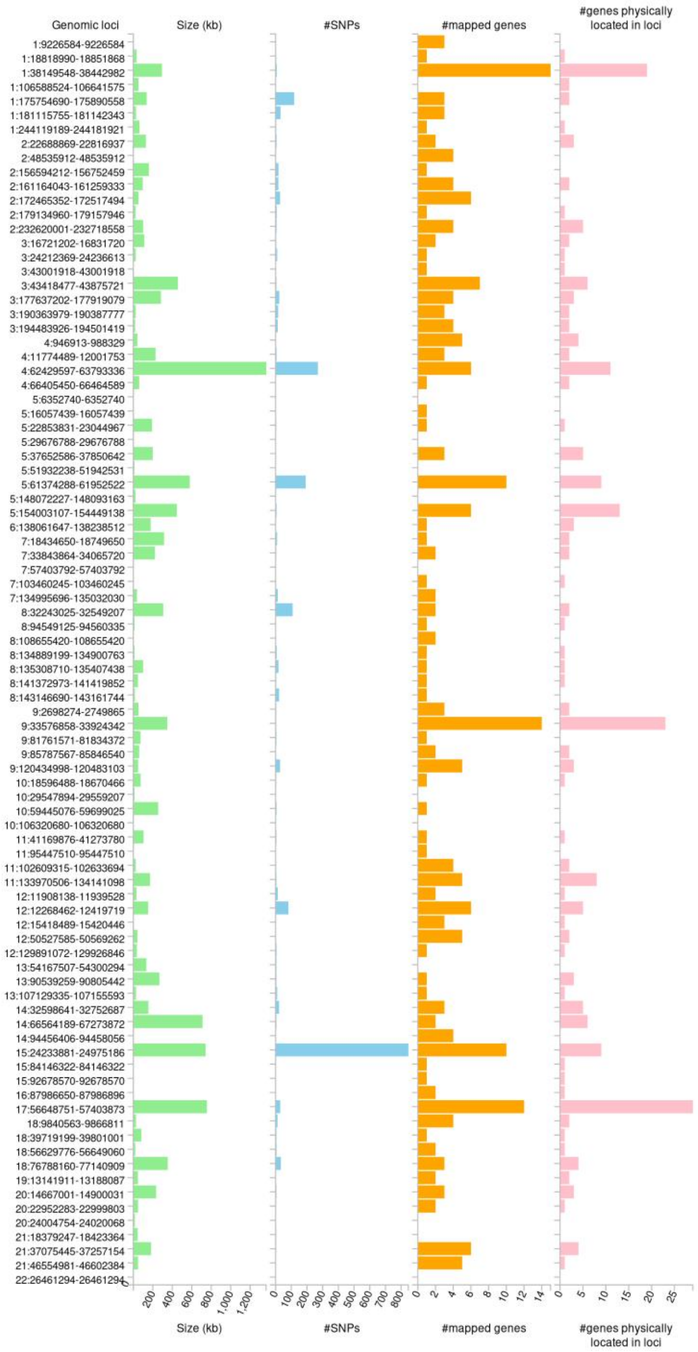

Supplemental Figure 2. Significant genomic loci associated with plasma potassium levels. The bar plot shows the significant genomic loci with their size in kilobases (kb), the number of SNPs and of mapped genes within each locus, and the number of genes physically located in each locus.

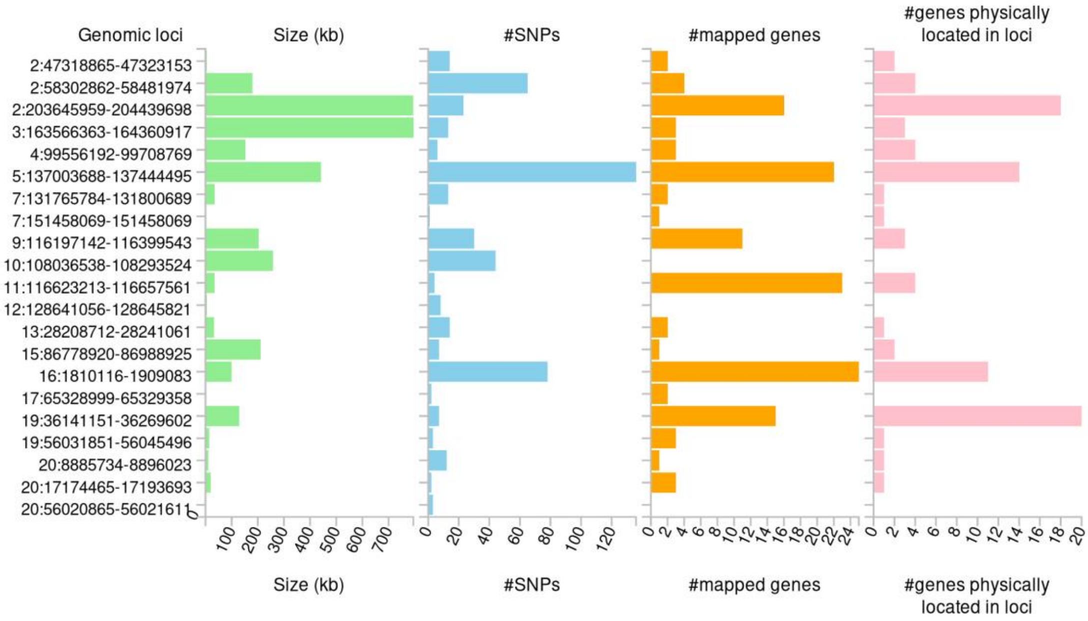

Supplemental Figure 3. Significant genomic loci associated with plasma sodium levels. The bar plot shows the significant genomic loci with their size in kilobases (kb), the number of SNPs and of mapped genes within each locus, and the number of genes physically located in each locus.

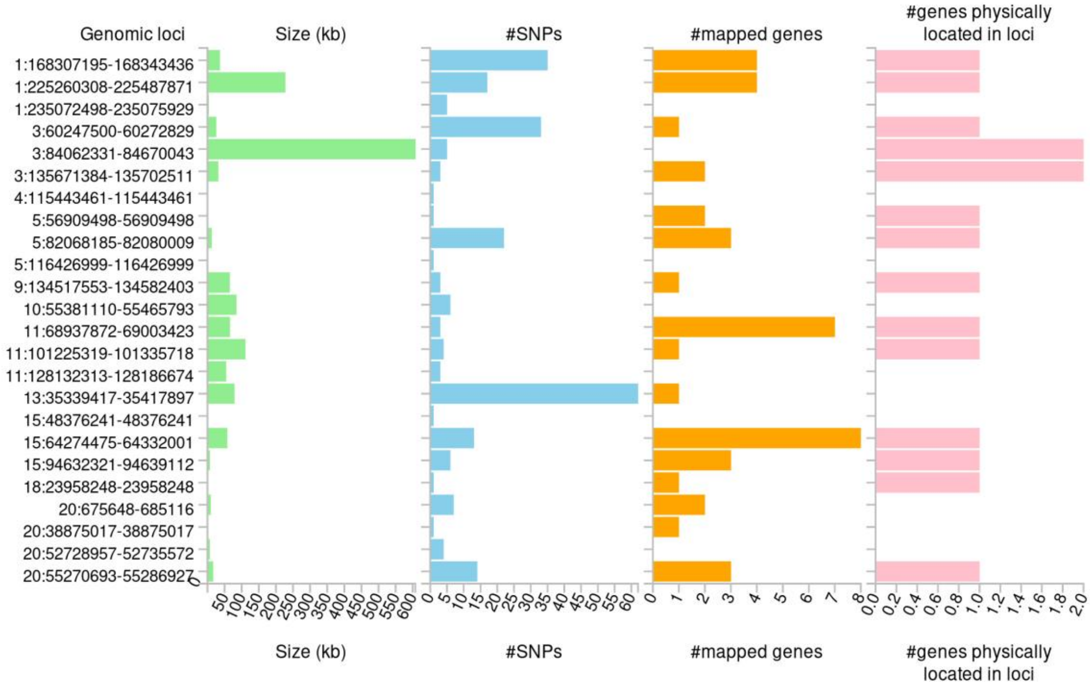

Supplemental Figure 4. Gene sets tissue expression analysis for plasma sodium levels.

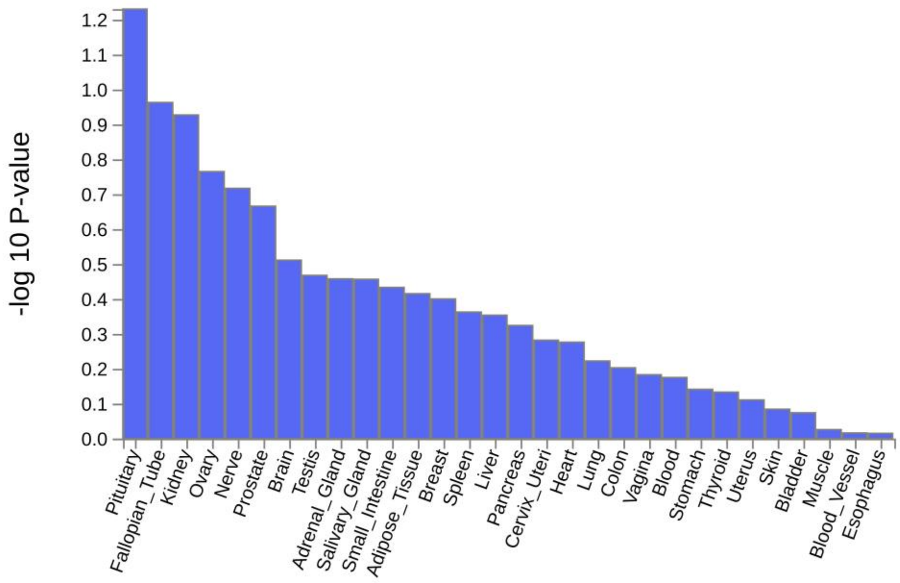

Supplemental Figure 5. MAGMA expression analysis on the specific tissue types from the Genotype-Tissue Expression portal for plasma chloride levels. Bar plots from the expression level across the 53 specific tissue types in the GTEx portal observed for plasma chloride levels.

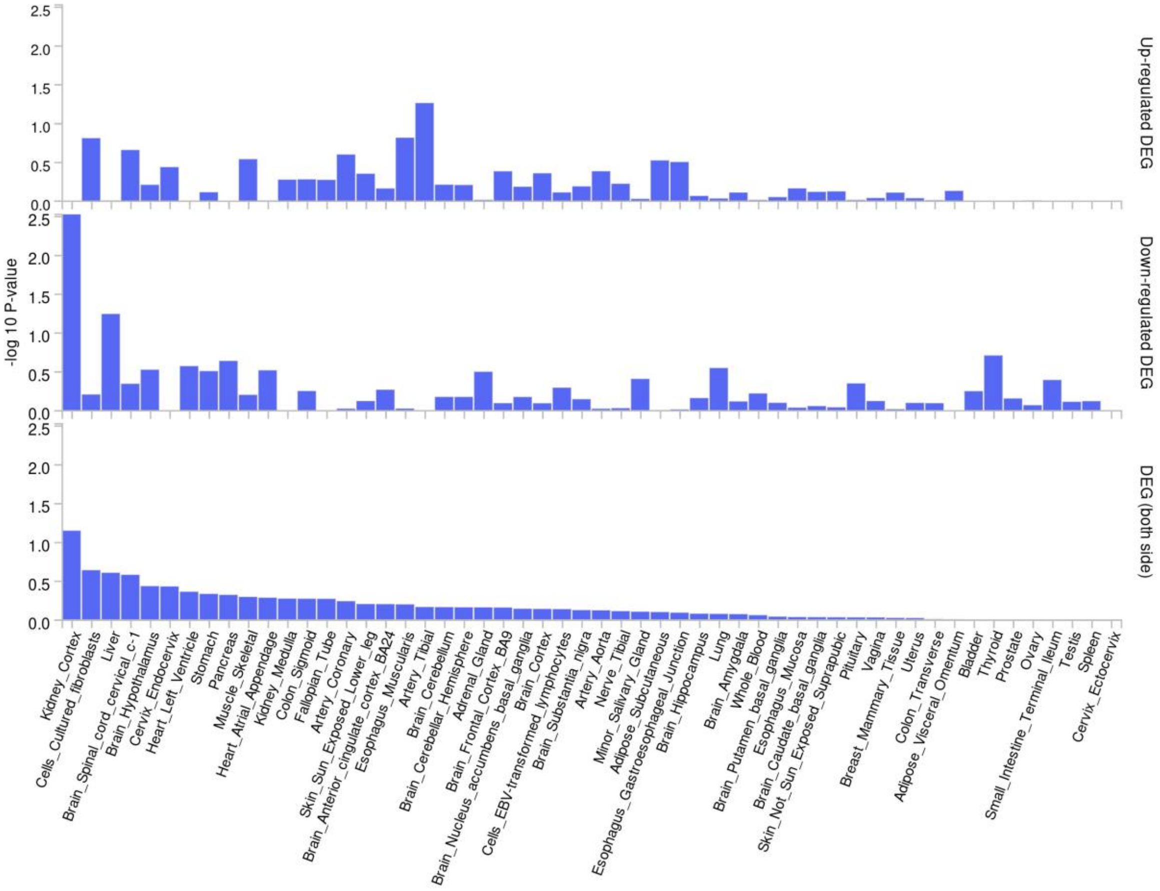

Supplemental Figure 6. MAGMA expression analysis on the specific tissue types from the Genotype-Tissue Expression portal for plasma potassium levels. Bar plots from the expression level across the 53 specific tissue types in the GTEx portal observed for plasma potassium levels. Significantly enriched tissue (Bonferroni-adjusted p-value < 0.05) are reported with red bars.

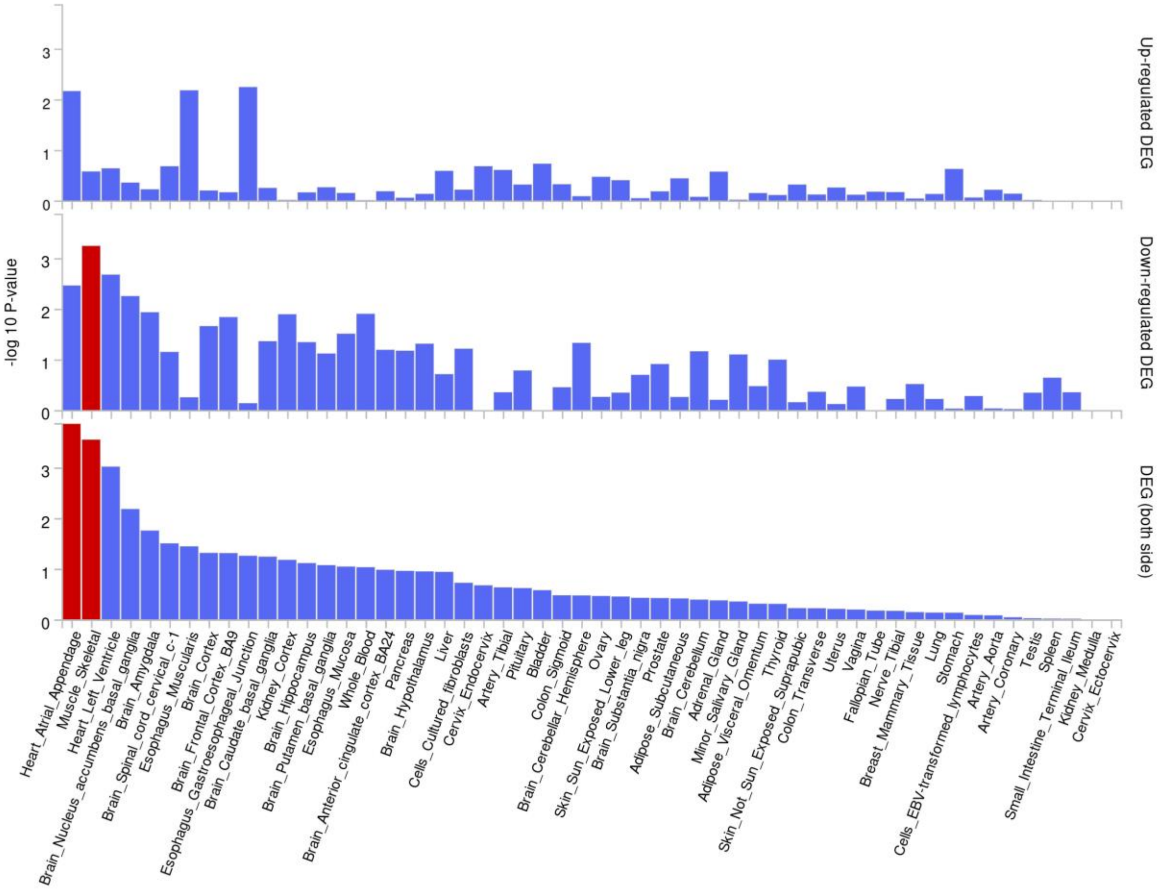

Supplemental Figure 7. MAGMA expression analysis on the specific tissue types from the Genotype-Tissue Expression portal for plasma sodium levels. Bar plots from the expression level across the 53 specific tissue types in the GTEx portal observed for plasma sodium levels.

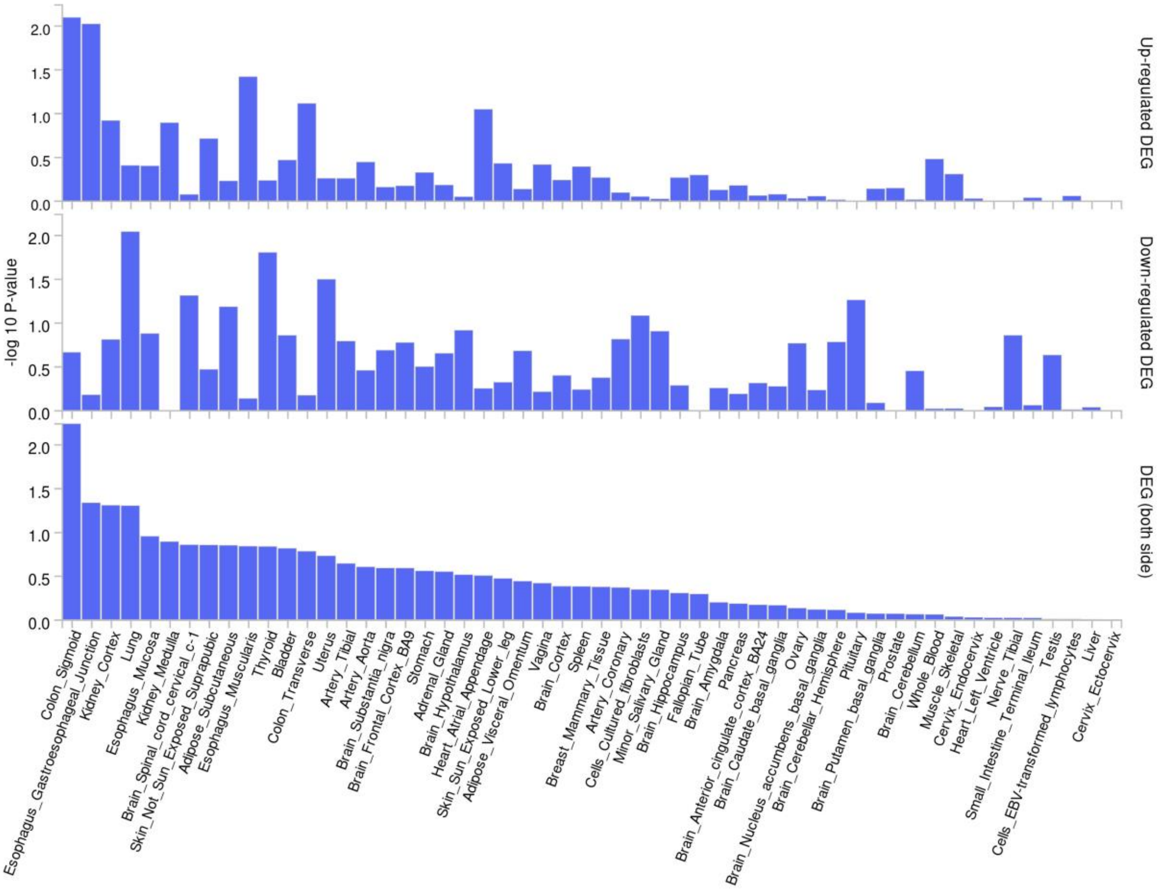

Supplement: Supplementary file 1 [file gfag069_supplemental_files.zip › SupplementalMaterials.pdf]
